# Machine Learning Models for Prediction of Maternal Hemorrhage and Transfusion: Model Development Study

**DOI:** 10.2196/52059

**Published:** 2024-02-05

**Authors:** Homa Khorrami Ahmadzia, Alexa C Dzienny, Mike Bopf, Jaclyn M Phillips, Jerome Jeffrey Federspiel, Richard Amdur, Madeline Murguia Rice, Laritza Rodriguez

**Affiliations:** 1 Division of Maternal-Fetal Medicine Department of Obstetrics and Gynecology George Washington University Washington, DC United States; 2 Division of Maternal-Fetal Medicine Department of Obstetrics and Gynecology Inova Health System Falls Church, VA United States; 3 The George Washington University School of Medicine and Health Sciences, Washington DC, DC United States; 4 Lister Hill National Center for Biomedical Communications U.S. National Library of Medicine Bethesda, MD United States; 5 Division of Maternal-Fetal Medicine Department of Obstetrics and Gynecology Duke University Durham, NC United States; 6 Medical Faculty Associates The George Washington University School of Medicine and Health Sciences Washington, DC United States; 7 George Washington University Biostatistics Center Washington, DC United States

**Keywords:** postpartum hemorrhage, machine learning, prediction, maternal, predict, predictive, bleeding, hemorrhage, hemorrhaging, birth, postnatal, blood, transfusion, antepartum, obstetric, obstetrics, women's health, gynecology, gynecological

## Abstract

**Background:**

Current postpartum hemorrhage (PPH) risk stratification is based on traditional statistical models or expert opinion. Machine learning could optimize PPH prediction by allowing for more complex modeling.

**Objective:**

We sought to improve PPH prediction and compare machine learning and traditional statistical methods.

**Methods:**

We developed models using the Consortium for Safe Labor data set (2002-2008) from 12 US hospitals. The primary outcome was a transfusion of blood products or PPH (estimated blood loss of ≥1000 mL). The secondary outcome was a transfusion of any blood product. Fifty antepartum and intrapartum characteristics and hospital characteristics were included. Logistic regression, support vector machines, multilayer perceptron, random forest, and gradient boosting (GB) were used to generate prediction models. The area under the receiver operating characteristic curve (ROC-AUC) and area under the precision/recall curve (PR-AUC) were used to compare performance.

**Results:**

Among 228,438 births, 5760 (3.1%) women had a postpartum hemorrhage, 5170 (2.8%) had a transfusion, and 10,344 (5.6%) met the criteria for the transfusion-PPH composite. Models predicting the transfusion-PPH composite using antepartum and intrapartum features had the best positive predictive values, with the GB machine learning model performing best overall (ROC-AUC=0.833, 95% CI 0.828-0.838; PR-AUC=0.210, 95% CI 0.201-0.220). The most predictive features in the GB model predicting the transfusion-PPH composite were the mode of delivery, oxytocin incremental dose for labor (mU/minute), intrapartum tocolytic use, presence of anesthesia nurse, and hospital type.

**Conclusions:**

Machine learning offers higher discriminability than logistic regression in predicting PPH. The Consortium for Safe Labor data set may not be optimal for analyzing risk due to strong subgroup effects, which decreases accuracy and limits generalizability.

## Introduction

Maternal morbidity and mortality have been regarded as a reflection of health care quality nationwide. Among lower-income countries, postpartum hemorrhage (PPH) is typically the most common cause of maternal mortality and remains among the top causes in higher-income countries. In the United States, hemorrhage accounted for 11.0% of deaths between 2011 and 2016 [1-4]. To address maternal hemorrhage, maternal hemorrhage protocols have been implemented, which incorporate prospective PPH risk assessment to tailor PPH prophylactic and management approaches for patients’ individual risk profiles. However, these protocols are often based on observational studies that approximated the strength of associations with hemorrhage via logistic regression (LR) models and combined the results of multiple studies together in a linear fashion [5-7]. However, “standard” LR assumes that (1) there is a linear relationship between predictors and the log odds of outcomes and (2) there are independent relationships between predictors. Additionally, LR and related models often perform poorly with large numbers of included variables [8,9]. Consequently, current risk stratification models fail to accurately ascertain pregnant patients’ risk of hemorrhage [10]. Studies attempting to validate existing LR and related models have instead identified gaps in the efficacy of these models, as the majority of patients with PPH and transfusions were stratified in low or moderate risk groups [11,12].

Machine learning offers an advantage to current risk assessment methods through its ability to create a robust model based on larger numbers of predictors, with nonlinear relationships and interactions between variables included in analyses [13]. Our objective in this analysis was to create a validated prediction model using machine learning for postpartum hemorrhage and transfusion to optimize risk-based triage and inform policy makers and stakeholders who aim to further reduce maternal morbidity and mortality associated with hemorrhage.

## Methods

### Data Collection

**Figure 1:**
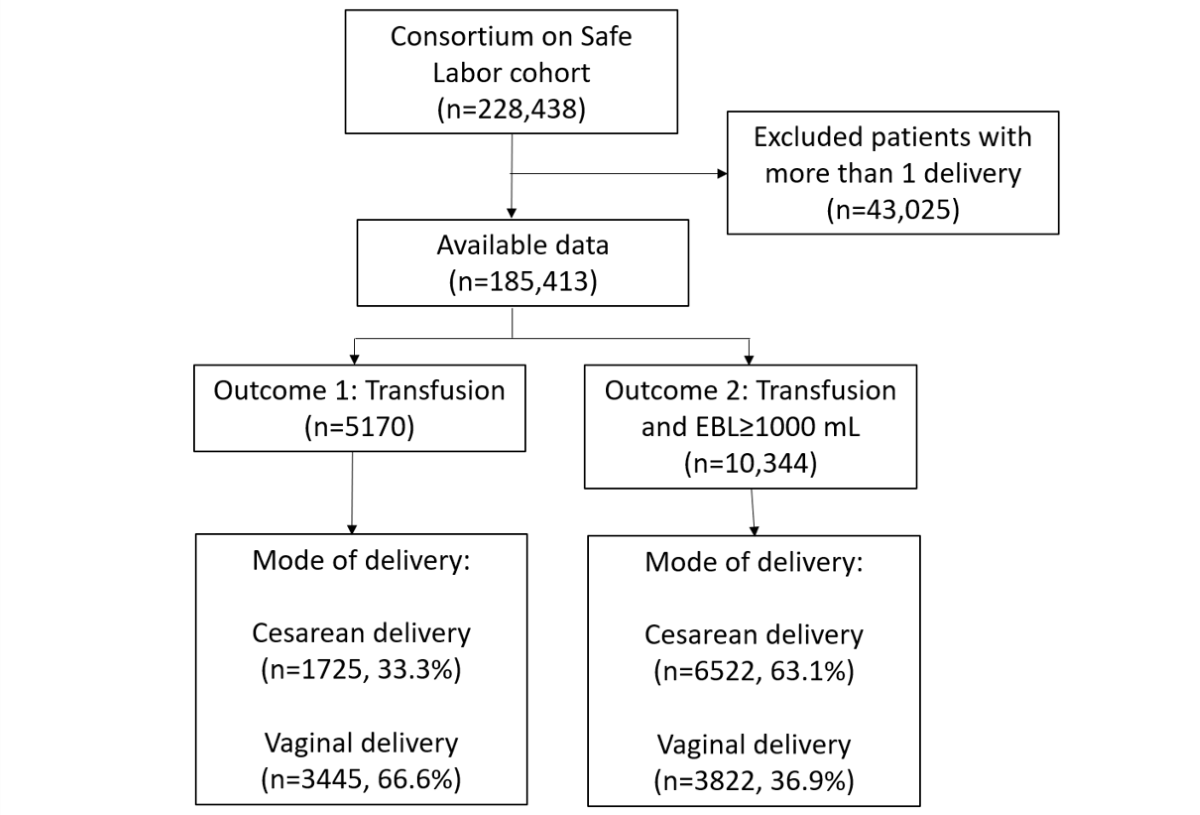
Flowchart of inclusion of women with transfusion or postpartum hemorrhage (or both).

Data for this analysis were extracted from the Consortium for Safe Labor (CSL) data set created by the Eunice Kennedy Shriver National Institute of Child Health and Human Development (NICHD). It includes antepartum, intrapartum, and postpartum medical histories of 224,438 women from 12 hospitals in the United States (Figure 1). Variables in this data set include maternal demographics, reproductive history, medical history, prenatal history of current pregnancy, labor admission assessment, labor progression, labor and delivery summary, maternal postpartum condition, and newborn information. For this database, data were extracted retrospectively from existing records for deliveries most recently occurring at each site. Data were extracted electronically using a method suitable to each hospital’s unique data systems. Data transfer and integrity were managed by a data coordinating center that created a central database. The data were deidentified and are available for research under request from the NICHD. Women with only 1 recorded pregnancy in the data set were included for data analysis; if women had more than 1 pregnancy during the study period, only the first one was used in the analysis. We selected maternal, fetal, and pregnancy variables as candidates to build the prediction model for transfusion risk.

### Missing Data

Machine learning methods are known to generate errors in the presence of missing values [14]. To avoid this, we imputed values as follows: categorical variables with missing and unknown values were assigned to an “unknown” category; continuous variables with missing and unknown values were coded to the median value. Continuous variables for maternal age and BMI were coded into ordinal categories (age of <20, between ≥20 and <40, between ≥40 and <45, and ≥45 years; BMI of ≤20, between >20 and ≤40, between >40 and ≤50, and >50 kg/m^2^). Imputing estimated blood loss (EBL) as the median value (350 mL) meant that missing values were assumed to be <1000 mL.

### Feature Selection

We used the Cramér V index of nominal association for variable selection [15]. Features were classified into antepartum and intrapartum variables. Two different prediction models were constructed: (1) an antenatal-only model intended to be used in the clinic setting to inform appropriate patient referral and (2) an intrapartum model that included both antepartum and intrapartum characteristics. Individual antepartum and intrapartum maternal variables included for model development are shown in the Multimedia Appendix 1.

### Outcomes

Separate models were constructed to predict 2 target outcomes. The primary outcome was a composite including all patients who received a transfusion of any blood product or had a PPH defined by documented blood loss of ≥1000 mL during or after delivery. Our secondary outcome was all patients who received transfusion of any blood product. Both blood loss of ≥1000 mL and blood transfusion are clinically significant metrics in obstetric care. Transfusion alone represents patients who are at risk for high maternal morbidity and mortality and is a clinically important metric to evaluate in isolation; hence, it was evaluated independently in a model as a secondary outcome.

### Data Analysis

For each of the 4 combinations of predictors and outcomes (for predictors, antepartum vs antepartum and intrapartum; for outcomes, transfusion and blood loss greater than a liter versus transfusion alone), the data were split so that 70% of the observations were used for training and 30% were used for testing, with both sets having the same outcome rate. We applied a number of methods, including LR, support vector machines (SVMs), multilayer perceptron (MLP), random forest (RF), and gradient boosting (GB), as well as deep learning algorithms including TensorFlow imbalanced (TFIM) and learned embedding (Emb). Hyperparameters were tuned for each algorithm using a customized grid search technique. The model performance for each combination of outcome and algorithm was measured using the Matthews correlation coefficient (MCC), area under the receiver operating characteristic curve (ROC-AUC), area under the precision/recall curve (PR-AUC), and modified F-score skewed toward recall (F2). A modified F2 score was chosen to minimize false negatives and thus maximize the identification of patients at high risk for bleeding and transfusion. Existing LR models and risk classification schemes perform poorly, and the majority of patients with hemorrhage or transfusion are misclassified as low risk. Misclassification of a “high risk” patient as “low risk” may have important clinical implications. Additionally, interventions can be implemented to minimize risk and enhance patient safety (eg, type and cross, multiple intravenous access sites, provider awareness, medications, etc). Models will then be evaluated for those with the highest positive predictive value (PPV) given these parameters. A model with the highest PPV will be clinically useful to identify a high-risk patient population without increasing the clinical burden on the hospital system or patient with the abovementioned interventions. Algorithms were processed and results were analyzed using Python (version 3.6; Python Software Foundation), Pandas (version 1.2; The Pandas Development Team), scikit-learn (version 0.24; scikit-learn Developers), and TensorFlow (version 2.2; Python Software Foundation).

The primary study objective was to identify the strongest set of pre- and intraoperative predictors of hemorrhage or transfusion and the strongest modeling technique. Secondary objectives included determining the level of agreement between metrics for model evaluation and the extent to which any technique produced results that are clinically useful. Given the heterogeneity of this data set derived from multiple institutions, a site-specific sensitivity analysis was performed.

### Ethical Considerations

This analysis was exempt from review by the George Washington University’s institutional review board (NCR202746).

## Results

Of 228,438 births included in the CSL cohort, we included 185,413 patients (Figure 1), having excluded patients with more than 1 delivery (n=43,025). Maternal age ranged from 11 to 58 (median 27) years; 32% (n=60,193) of the participants were publicly insured, 49% (n=90,466) were white non-Hispanic, 22% (n=41,780) were Black, and 17% (n=32,727) were Hispanic. Of the 185,413 women included in the analysis, 71% (n=131,130) had a vaginal delivery, and 29% (n=54,283) had a cesarean delivery. In total, 5170 (3%) women experienced the primary outcome of transfusion of any blood product, 5760 (3.11%) had a PPH defined by an estimated blood loss of ≥1000 mL, and 10,344 (6%) experienced the secondary composite outcome of transfusion or estimated blood loss of loss of ≥1000 mL. Additional demographic data are summarized in Multimedia Appendix 2.

After building the models in an iterative process, their performance in predicting both the primary and secondary outcomes was compared using a variety of metrics. The metrics ROC-AUC, PR-AUC, MCC, and F2, as well as sensitivity and specificity at a probability cut point of 50% are shown in Tables 1 and 2.

**Table 1:** Performance of machine learning and statistical models based on antepartum and intrapartum maternal variables at predicting transfusion or postpartum hemorrhage (or both). Primary outcome: blood transfusion or blood loss of ≥1 L.

Algorithm	True positives^a^, n	True negatives^a^, n	False positives^a^, n	False negatives^a^, n	Positive predictive value	Sensitivity	Specificity	ROC-AUC^b^	PR-AUC^c^	MCC^d^	F2^e^
GB^f^	50	6	318	626	0.135	0.889	0.663	0.833	0.210	0.260	0.419
RF^g^	50	6	339	605	0.138	0.857	0.641	0.830	0.204	0.261	0.409
Emb^h^	46	10	296	649	0.134	0.821	0.687	0.813	0.181	0.246	0.406
MLP^i^	49	7	335	609	0.127	0.875	0.645	0.808	0.149	0.245	0.402
TFIM^j^	48	8	323	619	0.129	0.861	0.655	0.822	0.194	0.245	0.403
SVM^k^	49	6	349	595	0.124	0.886	0.630	0.804	0.159	0.242	0.397
LR^l^	46	10	314	631	0.129	0.830	0.668	0.813	0.177	0.238	0.393

^a^Values are normalized per 1000, so they are easier to compare across different models; the actual N value is 55,624.

^b^ROC-AUC: area under the receiver operating characteristic curve.

^c^PR-AUC: area under the precision-recall curve.

^d^MCC: Matthews correlation coefficient.

^e^F2: modified F-score skewed toward recall.

^f^GB: gradient boosting.

^g^RF: random forest.

^h^Emb: learned embedding.

^i^MLP: multilayer perceptron.

^j^TFIM: TensorFlow imbalanced.

^k^SVM: support vector machine.

^l^LR: logistic regression.

**Table 2:** Performance of machine learning and statistical models based on antepartum and intrapartum maternal variables in predicting transfusion or postpartum hemorrhage (or both). Secondary outcome: blood transfusion.

Algorithm	True positives^a^, n	True negatives^a^, n	False positives^a^, n	False negatives^a^, n	Positive predictive value	Sensitivity	Specificity	ROC-AUC^b^	PR-AUC^c^	MCC^d^	F2^e^
GB^f^	24	4	235	737	0.093	0.866	0.758	0.860	0.111	0.234	0.325
RF^g^	25	3	251	721	0.090	0.887	0.742	0.862	0.107	0.232	0.319
Emb^h^	22	6	223	750	0.090	0.789	0.771	0.837	0.096	0.215	0.309
MLP^i^	24	4	237	735	0.091	0.849	0.756	0.845	0.095	0.227	0.318
TFIM^j^	24	4	240	732	0.091	0.859	0.753	0.855	0.111	0.229	0.319
SVM^k^	24	4	244	728	0.091	0.871	0.749	0.852	0.116	0.230	0.320
LR^l^	24	3	250	722	0.089	0.876	0.743	0.853	0.111	0.228	0.317

^a^Values are normalized per 1000, so they are easier to compare across different models; the actual N value is 55,624.

^b^ROC-AUC: area under the receiver operating characteristic curve.

^c^PR-AUC: area under the precision-recall curve.

^d^MCC: Matthews correlation coefficient.

^e^F2: modified F-score skewed toward recall.

^f^GB: gradient boosting.

^g^RF: random forest.

^h^Emb: learned embedding.

^i^MLP: multilayer perceptron.

^j^TFIM: TensorFlow imbalanced.

^k^SVM: support vector machine.

^l^LR: logistic regression.

**Figure 2:**
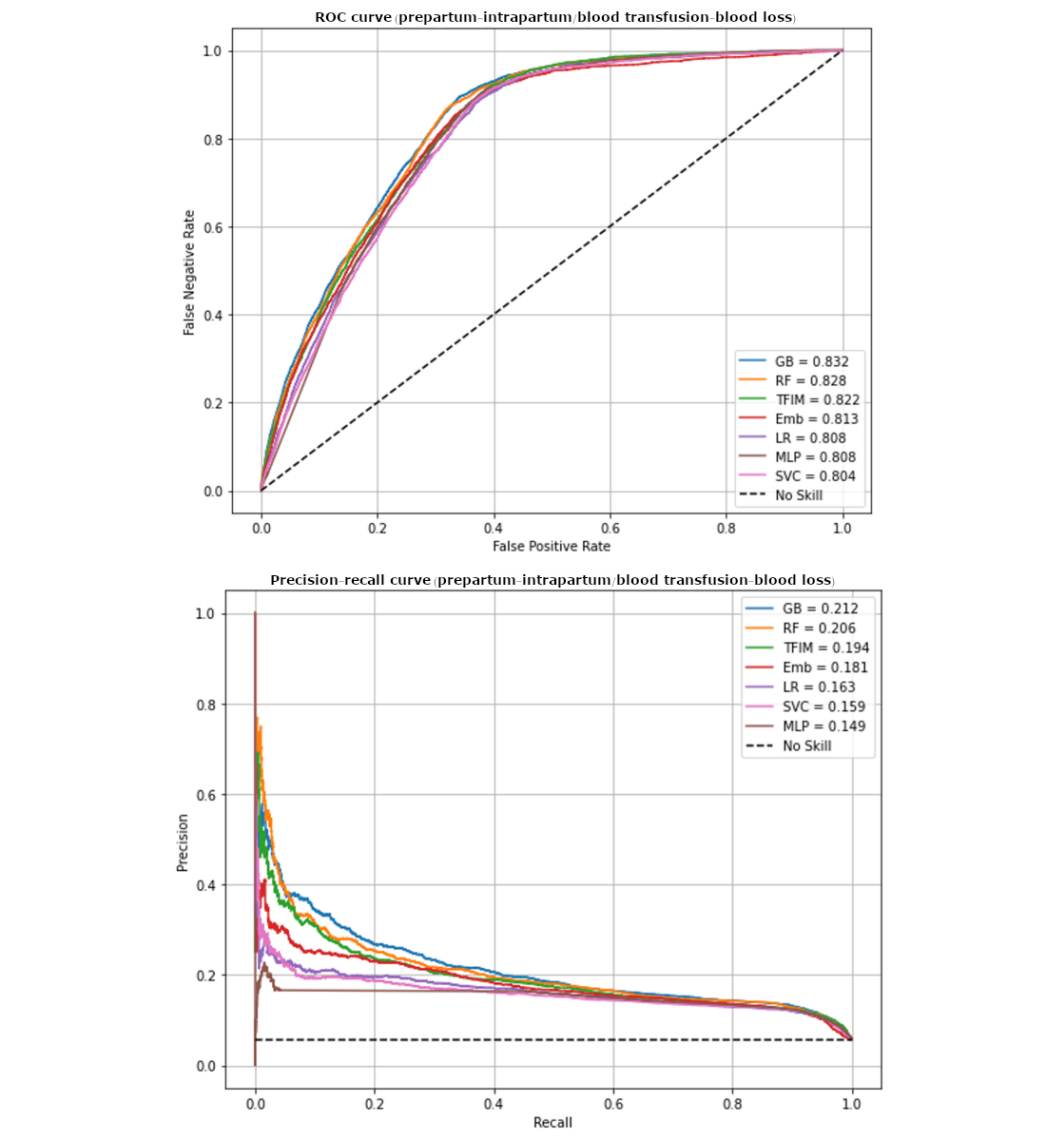
Receiver operating characteristic and precision/recall curves for different models using intrapartum maternal variables predicting transfusion or postpartum hemorrhage.

For both the primary and secondary outcomes, models developed using antepartum and intrapartum maternal variables (see Multimedia Appendix 1 for a list of variables) to predict the primary outcome performed better with higher PPVs than those solely using antepartum maternal variables (Multimedia Appendices 3 and 4). For the primary composite outcome, the machine learning technique GB using intrapartum maternal variables had the highest PPV (PR-AUC=0.21, 95% CI 0.20-0.22; ROC-AUC=0.83, 95% CI 0.828-0.838; Figure 2). For the secondary outcome of transfusion alone, there was little difference in model performance when comparing several performance metrics.

The remainder of our results focus on the model with the highest PPV: the intrapartum model (containing both antepartum and intrapartum variables) evaluating our primary outcome of a composite of blood loss of more than 1000 mL or transfusion. Both RF and GB had significantly higher PPVs for predicting the composite transfusion or PPH when compared with LR (PR-AUC=0.18, 95% CI 0.17-0.19; ROC-AUC=0.81, 95% CI 0.808-0.818).

**Figure 3:**
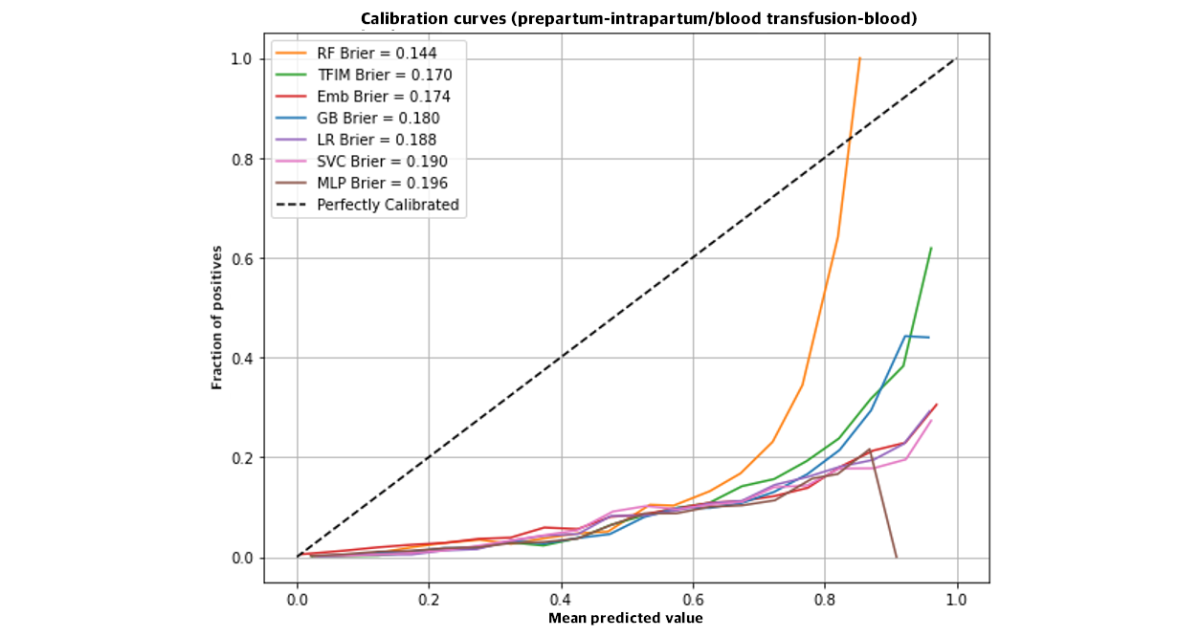
Calibration curves for models using intrapartum maternal variables to predict transfusion or postpartum hemorrhage (or both). Emb: learned embedding; GB: gradient boosting; LR: logistic regression; MLP: multilayer perceptron; RF: random forest; SVC: support vector machine; TFIM: TensorFlow imbalanced.

Figure 3 reveals the calibration curves for the models constructed with intrapartum maternal variables and predicting the transfusion-PPH composite. Calibration curves portray the predicted PPH risk versus the observed PPH rate across a range of predicted PPH values. There was better agreement between the models with a lower fraction of positives, and none of the models were able to reach the standard curve—for all models, the predicted PPH risk overestimated the observed PPH rate across the range of predicted values.

**Figure 4:**
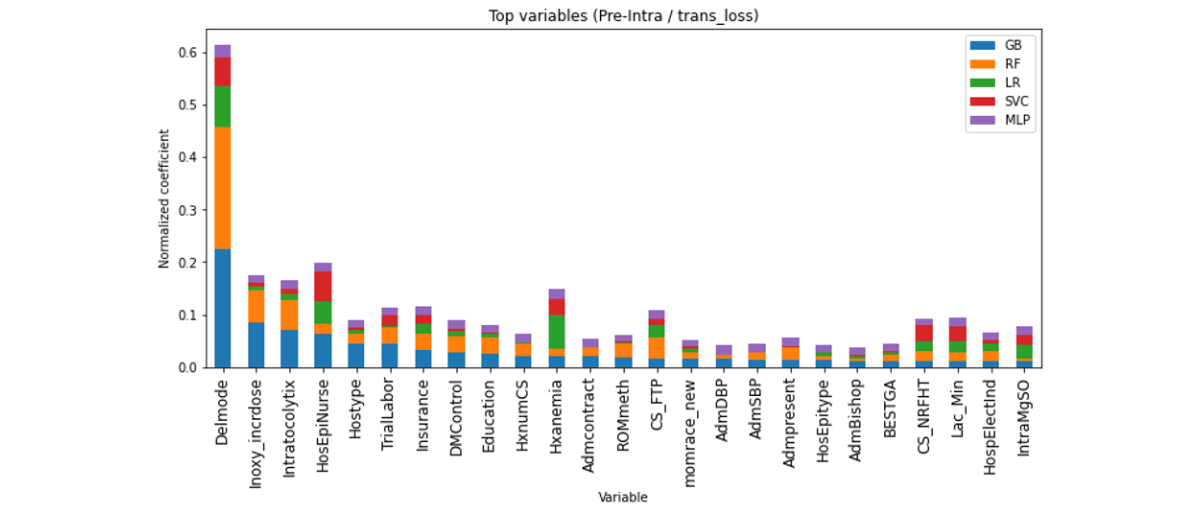
Top 25 predictors based on each model using intrapartum maternal factors predicting transfusion or postpartum hemorrhage (or both). GB: gradient boosting; LR: logistic regression; MLP: multilayer perceptron; RF: random forest; SVC: support vector machine.

Figure 4 displays the top 25 predictive variables included for model development using antepartum and intrapartum features for the prediction of the transfusion-PPH composite. As the machine learning GB model was the best performing model overall, the variables in Figure 4 are in order of variable importance within the GB model. The top 10 variables from most predictive rate to least predictive rate for intrapartum prediction of the transfusion-PPH composite using the GB model are mode of delivery, oxytocin incremental dose for labor (mU/minute), intrapartum tocolytic use, use of anesthesia nurse, hospital type, a trial of labor after prior cesarean delivery, insurance, most serious diabetes control, education, and history of prior cesarean sections. The results of the models for antepartum-only models are listed in Multimedia Appendix 3. The ROC-AUC and PR-AUC did not perform as well for the models using antepartum-only variables, though this was less obvious for the models predicting transfusion only (Multimedia Appendix 4). Of note, upon further sensitivity analysis, we also determined that some of the top variables in the model were site-specific (ie, oxytocin incremental dose for labor, intrapartum tocolytic use, use of anesthesia nurse, and hospital type) for transfusion outcomes specifically (data not included).

## Discussion

### Principal Findings

In this study, LR and machine learning techniques were analyzed and compared to develop prediction models for PPH and transfusions. We found that the machine learning techniques, particularly GB, performed best to predict PPH when PPH was defined as blood transfusion or blood loss of greater than 1 L. However, all prediction models had difficulties with calibration when predicting the rare outcome of transfusion alone.

### Clinical Implications

Risk assessment for PPH has been shown in a pre-post study to reduce rates of blood transfusion and PPH [16]. However, the risk stratification approaches most commonly used for PPH in the United States were developed and implemented on the basis of expert opinion, and subsequent validation studies have revealed the limitations of these tools [17,18]. Validation studies using the California Maternal Quality Care Collaborative (CMQCC) risk assessment tool found that while the tool generated populations with different rates of hemorrhage among those stratified to low, medium, and high-risk groups, the rate of PPH among women stratified in the high-risk group for PPH was only 22% [19]. Others have found that the AUC-ROC for the CMQCC and Association of Women's Health, Obstetric and Neonatal Nurses’ (AWHONN’s) tools for predicting severe PPH, defined by transfusion of at least 4 units packed red blood cells during postpartum period, were relatively modest at 0.77 and 0.69, respectively [20]. Furthermore, parameters that are included in PPH risk models based on univariate association with PPH risk may not be independent predictors when incorporated into multivariate models [20]. For these reasons, improvements in PPH risk models are a promising target for improving PPH care.

A previously published risk assessment for PPH using the CSL data set demonstrated exceptional model performance, but model performance was drastically lower in an external validation cohort [21,22]. This study augments the findings of these prior studies via incorporation of antepartum and intrapartum risk factors. Nonetheless, additional work is needed before such a model can be implemented in clinical practice. In particular, it will be important to develop prediction models that are implementable either through straightforward bedside data entry or can be automated via real-time data capture from electronic medical records, which are well validated in a variety of hospital settings, and ideally, which are paired with recommended risk-based interventions to reduce hemorrhage risk and mitigate the occurrence of hemorrhage. In our study, among the top predictors were variables that reflect patients’ access to care and resources, such as hospital type and insurance. This highlights the possible need for a layered prediction model, which may help stratify patients who may need to be transferred to a tertiary care center with more resources (using an antepartum model focusing on patient factors along with hospital factors to designate risk).

### Research Implications

For all the intrapartum methods that we tested for predicting transfusion or hemorrhage, the ROC-AUC values were greater than 0.80, which is often cited as a threshold indicating adequate discrimination. However, this conclusion is misleading because in a situation where incidence of the outcome is low (here, it was ~3% for transfusion or hemorrhage alone), the PPV, also known as “precision,” is likely to be quite low. Our precision for the best-performing model was ~13%, meaning that of those predicted to be positive for the outcome, 13% were positive and 87% were negative. This may be satisfactory for clinical uses where preventive interventions have very low cost (in terms of both financial cost and added risk to the patient) but would not be acceptable when the intervention is of higher risk or is more expensive. In this situation, the PR-AUC provided a more realistic measure of model quality. Precision/recall plots show PPV (aka precision) as a function of sensitivity (aka recall); thus, they account for true positives in positive predictions. In contrast, the ROC-AUC emphasizes specificity, which is likely to be very high when true positives are rare [23,24]. The metric with the largest difference between the best and worst-performing models is PR-AUC (0.16 vs 0.21). This metric could be used more frequently in modeling studies when the occurrence of the outcome of interest is ≤6%.

### Strengths and Limitations

The strengths of this study include the use of a large, national multicenter data set to develop a data-driven model that can predict PPH using antepartum and intrapartum factors using cutting-edge machine learning techniques. Furthermore, we considered both commonly used end points such as estimated blood loss greater than 1 L and clinically relevant end points such as transfusion; this led us to conclude that due to a less frequent occurrence and transfusion practice, variation made it more challenging to develop a reliable model for transfusion only.

Limitations of the study include the low reported precision of algorithms. Sensitivity is prioritized for prediction, as clinically missing PPH has more consequences than a false positive. Therefore, the algorithms are trained to be biased toward predicting positives resulting in lower false negative rates at the risk of higher false positive rates and decreased precision. As a result, as shown in the calibration plots, the models systematically overstate hemorrhage risk. In this study, the outcomes of interest were either a composite of transfusion or blood loss of ≥1 L or transfusion only. Our PPH definition was based on the American College of Obstetricians and Gynecologists’ reVITALize program’s definition of PPH as blood loss of ≥1 L or loss of blood with clinical signs of hypovolemia within 24 hours of delivery. This definition deviates from older traditional definitions that defined PPH as ≥500 mL for vaginal delivery and 1000 mL for cesarean delivery [25]. Therefore, clinical care could have been guided by older definitions, as the CSL data set was collected between 2002 and 2008 [21]. However, a strength of our study is the use of EBL rather than a clinical designation of PPH so that we only include patients who were designated to have an EBL above the current threshold for PPH, that is, 1000 mL. Beyond that, measures of EBL have been shown to be imprecise with low volumes overestimated and high volumes of blood loss underestimated [26]. Furthermore, transfusion was used as a proxy for PPH, and transfusion thresholds vary depending on the institution and provider. In addition, the machine learning algorithms are limited by the variables measured and accurately recorded in the data set.

### Conclusions

In conclusion, machine learning and data-driven statistical modeling may offer more objective and discriminative prediction of PPH based on individual antepartum and intrapartum patient features, compared to expert opinion, and may improve upon traditional regression models. This can increase the opportunity for precision medicine and improved clinical care to reduce the burden of PPH as a leading cause of maternal morbidity and mortality.

## Appendix

Multimedia Appendix 1 All antepartum and intrapartum variables were included for analysis for feature selection.

Multimedia Appendix 2 Overall Patient Characteristics.

Multimedia Appendix 3 Performance of machine learning and statistical models. The model included antepartum maternal features predicting transfusion and/or postpartum hemorrhage. Pre/Trans Loss. Footnote for table: aAlg=algorithm, bNTP=normalized true positive, cNFN=normalized false negative, dNFP=normalized false positive, eNTN=normalized true negative, fROC_AUC (receiver operator curve_area under the curve; 0.5 was considered no better than chance, greater than 0.5 to less than 0.7 poor, 0.7 to less than 0.8 acceptable, 0.8 to less than 0.9 excellent, 0.9 or greater outstanding), gPR_AUC (precision recall_area under the curve), hMCC=Matthews correlation coefficient, iF2= modified F-score skewed towards recall), jGradient boosting, kRandom forests, llearned embedding, mMulti-layer percepton, nTensorflow imbalanced, oSupport vector machines, plogistic regression.

Multimedia Appendix 4 Performance of machine learning and statistical models. The model included antepartum maternal features predicting transfusion of any blood products only. Pre/ Trans_yes Footnote: aAlg=algorithm, bNTP=normalized true positive, cNFN=normalized false negative, dNFP=normalized false positive, eNTN=normalized true negative, fROC_AUC (receiver operator curve_area under the curve; 0.5 was considered no better than chance, greater than 0.5 to less than 0.7 poor, 0.7 to less than 0.8 acceptable, 0.8 to less than 0.9 excellent, 0.9 or greater outstanding), gPR_AUC (precision recall_area under the curve), hMCC=Matthews correlation coefficient, iF2= modified F-score skewed towards recall), jGradient boosting, kRandom forests, llearned embedding, mMulti-layer percepton, nTensorflow imbalanced, oSupport vector machines, plogistic regression.

## Abbreviations

CSL: Consortium for Safe Labor
EBL: estimated blood loss
Emb: learned embedding
GB: gradient boosting
LR: logistic regression
MCC: Matthews correlation coefficient
MLP: multilayer perceptron
NICHD: Eunice Kennedy Shriver National Institute of Child Health and Human Development
PPH: postpartum hemorrhage
PPV: positive predictive value
PR-AUC: precision/recall area under the curve
RF: random forest
ROC-AUC: receiver operating characteristic area under the curve
SVM: support vector machine
TFIM: TensorFlow imbalanced
