# Supplementary material for: Machine Learning Models for Prediction of Maternal Hemorrhage and Transfusion: Model Development Study
Source: JMIR Bioinform Biotechnol. 2024 Feb 5;5:e52059. doi: 10.2196/52059 (PMC11135239; doi:10.2196/52059)
Supplement: Multimedia Appendix 1 [file bioinform_v5i1e52059_app1.docx]

| **Antepartum Variabels** | **Intrapartum Variables** |
| --- | --- |
| Activeherpes: Active genital herpes | Accrete: Placenta accrete |
| Admreason: Admission to L&D: Admission reason | Activeherpes: Active genital herpes |
| Alcohol: Prenatal History: Alcohol during  pregnancy | AdmBishop: Admission to L&D: Bishop score |
| Anteabruption: Prenatal History: Abruptio placentae | Admcervpos: Admission to L&D: Cervical position |
| Anteanemia: Prenatal History: Prenatal anemia | Admconsistency: Admission to L&D: Consistency |
| Anteasthma: Prenatal History: Prenatal asthma | Admcontract: Admission to L&D: Number of  contractions per 10 minutes |
| Antebleed3: Prenatal History: Bleeding in the 3rd Trimester | AdmDBP: Admission to L&D: Diastolic BP (mmHg) at admission |
| AnteCHBP: Prenatal History: Chronic hypertension | Admefface: Admission to L&D: Effacement |
| Prenatal History: Antepartum chorioamnionitis | Admpresent: Admission to L&D: Presentation |
| Antefetaldth: Prenatal History: Antepartum fetal death | Admreason: Admission to L&D: Admission reason |
| Antefetdistress: Prenatal History: Antepartum fetal distress | AdmSBP: Admission to L&D: Systolic BP (mmHg)  at admission |
| AnteGBS: Prenatal History: Antepartum Group B  Strep | Alcohol: Prenatal History: Alcohol during pregnancy |
| AnteGDM: Prenatal History: Gestational diabetes | Analgesia: Labor and delivery summary: Labor Analgesia |
| Antehospital: Prenatal History: Antenatal hospital admission | Anteabruption: Prenatal History: Abruptio placentae |
| AnteLGA: Prenatal History: Large for GA | Anteanemia: Prenatal History: Prenatal anemia |
| Anteprevia: Prenatal History: Placenta previa | Anteasthma: Prenatal History: Prenatal asthma |
| AnteSGA: Prenatal History: Intrauterine growth restriction | Antebleed3: Prenatal History: Bleeding in the 3rd Trimester |
| AnteSTD: Prenatal History: Sexually transmitted  disease infection | AnteCHBP: Prenatal History: Chronic hypertension |
| Antesteroid: Prenatal History: Antenatal Steroids | Antechorio: Antepartum chorioamnionitis |
| Antethrombo: Prenatal History: Thromboembolic  disorder | Antefetaldth: Prenatal History: Antepartum fetal  death |
| Antethyroid: Prenatal History: Prenatal thyroid disease | Antefetdistress: Prenatal History: Antepartum fetal distress |
| AnteUTI: Prenatal History: Antepartum UTI | AnteGBS: Prenatal History: Antepartum Group B  Strep |
| ART: Pregnancy by ART or drugs | AnteGDM: Prenatal History: Gestational diabetes |
| Cerclage: Cerclage | Antehospital: Prenatal History: Antenatal hospital admission |
| ChronicHBP: Chronic Hypertension | AnteLGA: Prenatal History: Large for GA |
| CPD: CPD | Anteprevia: Prenatal History: Placenta previa |
| CS_Elect: C Section - Elective | AnteSGA: Prenatal History: Intrauterine growth restriction |
| CS_Fetanom: C Section – Fetal indication or anomaly | AnteSTD: Prenatal History: Sexually transmitted  disease infection |
| CS_Fetmac: C Section – Fetal macrosomia | Antesteroid: Prenatal History: Antenatal Steroids |
| CS_HIV: C Section – HIV, Herpes active infection | Antethrombo: Prenatal History: Thromboembolic disorder |
| CS_HTNdis: C Section – Hypertensive Disease | Antethyroid: Prenatal History: Prenatal thyroid disease |
| CS_HxShoulder: C Section – Hx of Shoulder dystocia | AnteUTI: Prenatal History: Antepartum UTI |
| CS_Mult: C Section - Multiple gestation | ART: Pregnancy by ART or drugs |
| CS_Uscar: C Section - Previous uterine scar | Augment: Labor and Delivery summary: Method of labor augmentation |
| depression_comb: maternal chronic disease: Depression | BESTGA: Admission to L&D: Best estimate GA  (week) |
| Diabetes: Medical History: Pre-existing diabetes | Breech: Derived variable from chart: Breech  presentation |
| DMControl: Prenatal History: Most serious diabetes control | breech9: Maternal ICD9 collection: Breech  presentation (ICD-9 code 652.2) |
| Druguse: Prenatal History: Recreational drugs  during pregnancy | Cerclage: Prenatal History: Cerclage |
| Eclampsia: Prenatal History: Eclampsia | Chorio: Chorioamnionitis (ICD-9 code 658.4) |
| ECV: Prenatal History: External cephalic version | chorio9: Chorioamnionitis (ICD-9 code 658.4) |
| Education: Maternal demographic: Highest level of  education | ChronicHBP: Medical History: Chronic  hypertension |
| GestHBP: Prenatal History: Prenatal gestational hypertension | CPD; Prenatal History: CPD |
| gest_diab: Derived variable: Gestational diabetes (GDM) | CS_Breech: C Section - Breech or other  malpresentation |
| gest_htn: Gestational hypertension | CS_Chorio: C Section - Chorioamnionitis |
| gi_disease9: Maternal ICD9 collection: maternal chronic disease Gastrointestinal disease (ICD-9 codes 555, 556, 557, 558) | CS_Elect: C Section - Elective |
| GI_disease_comb: maternal chronic disease: Gastrointestinal disease | CS_Emerg: C Section - Emergency |
| Heart_disease_comb: maternal chronic disease, heart disease | CS_Fetanom: C Section - Fetal indication or anomaly |
| high_Age | CS_Fetmac: C Section - Fetal macrosomia |
| high_BMI | CS_Fforceps: C Section - Failed trial forceps or  vacuum |
| high_Gravidity | CS_Finduct: C Section - Failed induction |
| high_height | CS_FTP: C Section - Failure to progress/  cephalopelvic disproportion |
| HIV: Medical History: HIV | CS_FVBAC: C Section - Failed VBAC |
| HospElectCS: Elective C-Section prior to 41 weeks | CS_HIV: C Section - HIV, Herpes active lesions |
| HospElectInd: Elective induction prior to 41 weeks | CS_HTNdis: C Section - Hypertensive disease |
| Hostype: Type of hospital | CS_HxShoulder: C Section - Hx of Shoulder  dystocia |
| Hxanemia: Medical History: Anemia | CS_Mult: C Section - Multiple gestation |
| Hxasthma: Medical History: Asthma | CS_NRFHT: C Section - Non-reasurring fetal  testing/Fetal distress |
| Hxcsection: Reproductive history: Hx of Prior C-Section | CS_Other: C Section - Other |
| Hxdepression: Medical History: Depression | CS_Shoulder: C Section - Shoulder dystocia |
| HxGIdis: Medical History: Gastrointestinal disorder | CS_Uscar: C Section - Previous uterine scar |
| Hxheartdis: Medical History: Heart disease | Dehiscence: Labor and Delivery summary:  uterine dehiscence |
| Hxmacrosomia: Reproductive history: Hx of Macrosomia | Delfetalpos: Labor and Delivery summary:  Fetal position |
| HxnumCS: Reproductive history: Prior C-Sections | Delmode: Labor and Delivery summary:  Mode of Delivery |
| Hxpreterm: Reproductive history: Hx of Preterm Birth | depression_comb: Derived variable, maternal  chronic disease: Depression |
| Hxrenaldis: Medical History: Renal disease | Diabetes: Medical History: Pre-existing diabetes |
| Hxseizure: Medical History: Seizure | Dilat_lst: Repeated Measures: Dilation of 1st exam |
| HxSTD: Medical History: STD | DMControl: Prenatal History: Most serious diabetes control |
| Hxstillbirth: Reproductive history: Hx of Prior Still  Births | Druguse: Prenatal History: Recreational drugs during pregnancy |
| Hxthyroid: Medical History: Thyroid | Eclamp: Derived variable: Eclampsia |
| Hypertyro: Maternal hyperthyroidism (ICD-9 codes 242) | Eclampsia: Prenatal History: Eclampsia |
| Hyp_ace: Hypertensives: ACE Inhibitors | ECV: Prenatal History: External cephalic version |
| Hyp_alpha: Hypertensives: Alpha-blockers | Education: Maternal demographic: Highest level of  education |
| Hyp_beta: Hypertensives: Beta-blockers | Episiotomy: Labor and Delivery summary:  Episiotomy |
| Hyp_meth: Hypertensives: Methyldopa (Aldomet) | FSE: Labor and Delivery summary: Fetal Salp  electrode |
| iatro_hypo: Maternal iatrogenic hypothyroidism (ICD-9 codes 243, 244.0-244.3) | Gamethod: Admission to L&D: Method to  determine GA |
| ImposedPE: Prenatal History: Pre/eclampsia  superimposed on hypertension | GestHBP: Prenatal gestational hypertension |
| Incompetence: Prenatal History: Cervical  incompetence | gest_diab: Derived variable: Gestational diabetes (GDM) |
| Ind_anomaly: Indication for labor induction: Fetal  anomaly | gest_htn: Gestational hypertension |
| Ind_Chorio: Indication for labor induction:  Chorioamnionitis | gi_disease9: maternal chronic disease Gastrointestinal disease (ICD-9 codes 555, 556, 557, 558) |
| Ind_elect: Indication for labor induction: Elective  induction | GI_disease_comb: maternal chronic disease: Gastrointestinal disease |
| Ind_Fcomp: Indication for labor induction: Fetal  compromise | Heart_disease_comb: maternal chronic disease, heart disease |
| Ind_Fmacro: Indication for labor induction:  Suspected fetal macrosomia | high_Age |
| Ind_HTN: Indication for labor induction: Maternal  hypertensive disorders | high_BMI |
| Ind_Hxfetal: Indication for labor induction: Previous  history of fetal indications | high_Gravidity |
| Ind_HxMat: Indication for labor induction: History of  maternal indications | high_height |
| Ind_Mcond: Indication for labor induction: Maternal  condition | HIV |
| Ind_Postdate: Indication for labor induction:  Postdate (as determined by physician) | HosEpiNurse: Epidural anethesia: Use of anesthesia nurse |
| Ind_Still: Indication for labor induction: Stillbirth | HosEpitype: Epidural anethesia: Type of epidural |
| Ind_Vbleed: Indication for labor induction: Vaginal  bleeding | HospElectCS: Elective C-Section prior to 41 weeks |
| Insurance: Maternal demographic: Insurance type | HospElectInd: Elective induction prior to 41 weeks |
| iufd9: Intrauterine fetal death (ICD-9 code 656.4) | Hostype: Type of hospital |
| Marital: Maternal demographic: Marital status | Hxanemia; Medical History: Anemia |
| momrace_new: Maternal race, some missing data  replaced with data from repeat pregnancies | Hxasthma: Medical History: Asthma |
| MthInd_Mec: Method of induction: Mechanical | Hxcsection: Reproductive history: Hx of Prior C-Section |
| MthInd_Miso: Method of induction: Cytotec/  Misoprostol/PGE1 | Hxdepression: Medical History: Depression |
| MthInd_PGE2: Method of induction: Other  prostaglandins | HxGIdis: Medical History: Gastrointestinal disorder |
| no_TD: Maternal ICD-9 collection: No thyroid disease | Hxheartdis: Medical History: Heart disease |
| Oligo: Prenatal History: Oligohydramnios | Hxmacrosomia: Reproductive history: Hx of Macrosomia |
| other_TD: Other thyroid diseases (ICD-9 codes 240, 241, 193, 226, 245, 246) | HxnumCS: Reproductive history: Prior C-Sections |
| Parity: Reproductive history: Parity | Hxpreterm: Reproductive history: Hx of Preterm Birth |
| Polyhydramnios: Prenatal History: Polyhydramnios | Hxrenaldis: Medical History: Renal disease |
| PostHBP: Maternal Postpartum: HTN/preeclampsia | Hxseizure: Medical History: Seizure |
| Preeclampsia: Prenatal History: Preeclampsia/HELLP | HxSTD: Medical History: STD |
| prelaborCD: Derived variable from chart: Prelabor cesarean section | Hxstillbirth: Reproductive history: Hx of Prior Still Births |
| pre_diab: Derived variable: maternal diabetes | Hxthyroid: Medical History: Thyroid |
| pre_PROM | Hypertyro: Maternal hyperthyroidism (ICD-9 codes 242) |
| prim_hypo: Primary hypothyroidism (ICD-9 code 244.9, 244) | Hyp_ace: Hypertensives: ACE Inhibitors |
| renal_disease9: maternal chronic disease Renal disease (ICD-9 codes 585, 586, 588, 646.2) | Hyp_alpha: Hypertensives: Alpha-blockers |
| renal_disease_comb: maternal chronic disease: kidney disease | Hyp_any: Hypertensives: Any medication |
| Rhincompat: Prenatal History: Rh incompatability | Hyp_beta: Hypertensives: Beta-blockers |
| Smoke: Prenatal History: Smoking during  pregnancy | Hyp_calc: Hypertensives: Calcium Channel  Blockers |
| no_TD: Maternal ICD-9 collection: No thyroid disease | Hyp_clon: Hypertensives: Clonidine (Catapres) |
| ThreatenedPB: Prenatal History: Threatened preterm birth | Hyp_hydr: Hypertensives: Hydralazine |
| threatpb9: Threatened preterm birth (ICD-9 code 644.0) | Hyp_meth: Hypertensives: Methyldopa (Aldomet) |
| UnspecHBP: Prenatal History: Unspecified hypertension | Hyp_othr: Hypertensives: Others |
| Uscar: Derived variable: previous uterine scar | iatro_hypo: Maternal iatrogenic hypothyroidism (ICD-9 codes 243, 244.0-244.3) |
| version9: Successful external version of malpresentation (ICD-9 code 652.1) | ImposedPE: Pre/eclampsia superimposed on hypertension |
|  | Incompetence: Prenatal History: Cervical incompetence |
|  | Induction: Labor and Delivery summary: Labor induction |
|  | Ind_anomaly: Indication for labor induction: Fetal anomaly |
|  | Ind_Chorio: Indication for labor induction: Chorioamnionitis |
|  | Ind_elect: Indication for labor induction: Elective induction |
|  | Ind_Fcomp: Indication for labor induction: Fetal  compromise |
|  | Ind_Fmacro: Indication for labor induction:  Suspected fetal macrosomia |
|  | Ind_HTN: Indication for labor induction: Maternal  hypertensive disorders |
|  | Ind_Hxfetal: Indication for labor induction: Previous  history of fetal indications |
|  | Ind_HxMat: Indication for labor induction: History of  maternal indications |
|  | Ind_Mcond: Indication for labor induction: Maternal  condition |
|  | Ind_Postdate: Indication for labor induction:  Postdate (as determined by physician) |
|  | Ind_Still: Indication for labor induction: Stillbirth |
|  | Ind_Unkn: Indication for labor induction: Unknown |
|  | Ind_Vbleed: Indication for labor induction: Vaginal  bleeding |
|  | Inoxy_incrdose: Institution Oxytocin Increment  dose (mU/min) |
|  | Insurance: Maternal demographic: Insurance type |
|  | Intrachorio: Labor and Delivery summary:  Chroiamnionitis |
|  | Intrafetdistress: Labor and Delivery summary:  Intrapartum fetal distress |
|  | Intrafever: Labor and Delivery summary: Fever |
|  | IntraMgSO: Labor and Delivery summary:  magnesium sulfate |
|  | Intratocolytix: Labor and Delivery summary: Intra  tocolytics |
|  | intra_abruptio |
|  | intra_previa |
|  | iufd9: Intrauterine fetal death (ICD-9 code 656.4) |
|  | IUPC: Labor and Delivery summary: IUPC |
|  | Lac_Cerv: Lacerations: Cervical |
|  | Lac_Majoth: Lacerations: Other Major (Sulcus,  Vaginal wall) |
|  | Lac_Majperi: Lacerations: Major Perineal (3rd and  4th degree) |
|  | Lac_Min: Lacerations: Minor (perineal 1st/2nd  degres,labial, periurethral,clitoral,abrasion) |
|  | Lac_None: Lacerations: None |
|  | Lac_Other: Lacerations: Other |
|  | Malpresentation9: Other malpresentation of the  fetus (ICD-9 codes 652, 652.3- 652.9) |
|  | Marital: Maternal demographic: Marital status |
|  | Meconium: Labor and Delivery summary:  Meconium stain |
|  | momrace_new: Maternal race, some missing data  replaced with data from repeat pregnancies |
|  | Momseizure: Labor and Delivery summary:  maternal seizure |
|  | MthInd_AROM: Method of induction: Amniotomy/  AROM |
|  | MthInd_Mec: Method of induction: Mechanical |
|  | MthInd_Miso: Method of induction: Cytotec/  Misoprostol/PGE1 |
|  | MthInd_Oxy: Method of induction: Oxytocin/Pitocin |
|  | MthInd_PGE2: Method of induction: Other  prostaglandins |
|  | M_pre: Mild preeclampsia |
|  | no_TD: No thyroid disease |
|  | Oligo: Prenatal History: Oligohydramnios |
|  | Operative: Labor and Delivery summary: Operative delivery |
|  | other_TD: Other thyroid diseases (ICD-9 codes 240, 241, 193, 226, 245, 246) |
|  | Parity: Reproductive history: Parity |
|  | Polyhydramnios: Prenatal History: Polyhydramnios |
|  | PostHBP: Maternal Postpartum: HTN/  preeclampsia |
|  | Preeclampsia: Prenatal History: Preeclampsia/HELLP |
|  | prelaborCD: Derived variable from chart: Prelabor cesarean section |
|  | Presentdel: Labor and Delivery summary: Fetal  Presentation at Delivery |
|  | pre_diab: Derived variable: maternal diabetes |
|  | pre_PROM |
|  | prim_hypo: Primary hypothyroidism (ICD-9 code 244.9, 244) |
|  | Prolapse: Labor and Delivery summary: Cord prolapse |
|  | renal_disease9: maternal chronic disease Renal disease (ICD-9 codes 585, 586, 588, 646.2) |
|  | renal_disease_comb: maternal chronic disease: kidney disease |
|  | Rhincompat: Prenatal History: Rh incompatability |
|  | ROM: PROM, exclusive of spontaneous labor |
|  | ROMmeth: Labor and Delivery summary: Method of ROM |
|  | SE_pre: Superimposed preeclampsia |
|  | Shoulder: Labor and Delivery summary: Shoulder  dystocia |
|  | Smoke: Prenatal History: Smoking during  pregnancy |
|  | Spontlabor: Spontaneous labor |
|  | TD_nos: Unspecified thyroid disease during pregnancy |
|  | ThreatenedPB: Threatened preterm birth |
|  | threatpb9: Threatened preterm birth (ICD-9 code 644.0) |
|  | TrialLabor: Trial of Labor (defined as Vag  deliveries or C-Section with 2 records in repeated  measures database) |
|  | UnspecHBP: Prenatal History: Unspecified  hypertension |
|  | Urupture: Labor and Delivery summary: uterine rupture |
|  | Uscar: Derived variable: previous uterine scar |
|  | version9: Successful external version of malpresentation (ICD-9 code 652.1) |
|  | Vertex: Derived variable from chart: vertex  presentation |
