# Supplementary material for: Machine Learning Models for Prediction of Maternal Hemorrhage and Transfusion: Model Development Study
Source: JMIR Bioinform Biotechnol. 2024 Feb 5;5:e52059. doi: 10.2196/52059 (PMC11135239; doi:10.2196/52059)
Supplement: Multimedia Appendix 2 [file bioinform_v5i1e52059_app2.docx]

| **Characteristics** |  | **Number** | **Percentage** |
| --- | --- | --- | --- |
| ***Mode of Delivery*** | | | |
|  | Vaginal | 117640 | 63.4 |
|  | Vaginal-assisted | 9126 | 4.9 |
|  | VBAC | 4364 | 2.4 |
|  | Cesarean | 32991 | 17.8 |
|  | Cesarean-repeat | 21292 | 11.5 |
| ***Maternal Race*** | | | |
|  | Non-Hispanic White | 90466 | 48.8 |
|  | Non-Hispanic Black | 41780 | 22.5 |
|  | Hispanic | 32727 | 17.7 |
|  | Asian/ Pacific Islander | 7719 | 4.2 |
|  | Multi-racial | 316 | 0.2 |
|  | Other Race | 12405 | 6.7 |
| ***Age (year)*** | | | |
|  | <20 | 17242 | 9.3 |
|  | 20- 40 | 162006 | 87.4 |
|  | 40- 45 | 5972 | 3.2 |
|  | >45 | 193 | 0.1 |
| ***BMI*** | | | |
|  | <=20 | 19325 | 10.4 |
|  | 20- 40 | 161785 | 87.3 |
|  | 40- 50 | 3675 | 2.0 |
|  | >50 | 628 | 0.3 |
| ***Education*** | | | |
|  | Less than High School Diploma | 12014 | 6.5 |
|  | High School Diploma | 18709 | 10.1 |
|  | More than High School Diploma | 29647 | 16.0 |
|  | Unknown | 125043 | 67.4 |
| ***Insurance*** | | | |
|  | Public | 101657 | 54.83 |
|  | Private | 60193 | 32.46 |
|  | Hospital | 2276 | 1.23 |
|  | Other/Unknown | 21287 | 11.49 |
| ***Parity*** | Mean (SD)  Median (IQR) | - 1. (0.066)   1 (0,2) |  |
