# Supplementary material for: Machine Learning Models for Prediction of Maternal Hemorrhage and Transfusion: Model Development Study
Source: JMIR Bioinform Biotechnol. 2024 Feb 5;5:e52059. doi: 10.2196/52059 (PMC11135239; doi:10.2196/52059)
Supplement: Multimedia Appendix 3 [file bioinform_v5i1e52059_app3.docx]

| ^a^**^ALG^** | ^b^**NTP** | ^c^**NFN** | ^d^**NFP** | ^e^**NTN** | **precision** | **recall** | **specificity** | ^f^**ROC_AUC** | ^g^**PR_AUC** | ^h^**MCC** | ^i^**F2** |
| --- | --- | --- | --- | --- | --- | --- | --- | --- | --- | --- | --- |
| **^j^GB** |  |  |  |  |  |  |  |  |  |  |  |
|  | 44 | 12 | 319 | 625 | 0.121 | 0.787 | 0.662 | 0.786 | 0.176 | 0.214 | 0.374 |
| **^k^RF** |  |  |  |  |  |  |  |  |  |  |  |
|  | 44 | 12 | 338 | 606 | 0.116 | 0.793 | 0.642 | 0.777 | 0.163 | 0.206 | 0.365 |
| **^l^Emb** |  |  |  |  |  |  |  |  |  |  |  |
|  | 43 | 13 | 319 | 625 | 0.119 | 0.773 | 0.662 | 0.778 | 0.158 | 0.207 | 0.368 |
| **^m^MLP** |  |  |  |  |  |  |  |  |  |  |  |
|  | 41 | 15 | 299 | 645 | 0.121 | 0.739 | 0.683 | 0.771 | 0.164 | 0.204 | 0.366 |
| **^n^TFIM** |  |  |  |  |  |  |  |  |  |  |  |
|  | 39 | 17 | 279 | 665 | 0.122 | 0.692 | 0.705 | 0.762 | 0.155 | 0.195 | 0.357 |
| **^o^SVC** |  |  |  |  |  |  |  |  |  |  |  |
|  | 42 | 14 | 342 | 602 | 0.110 | 0.757 | 0.638 | 0.750 | 0.142 | 0.186 | 0.348 |
| **^p^LR** |  |  |  |  |  |  |  |  |  |  |  |
|  | 41 | 15 | 324 | 620 | 0.112 | 0.735 | 0.657 | 0.754 | 0.149 | 0.187 | 0.349 |
