# Supplementary material for: Machine Learning Models for Prediction of Maternal Hemorrhage and Transfusion: Model Development Study
Source: JMIR Bioinform Biotechnol. 2024 Feb 5;5:e52059. doi: 10.2196/52059 (PMC11135239; doi:10.2196/52059)
Supplement: Multimedia Appendix 4 [file bioinform_v5i1e52059_app4.docx]

| ^a^**Alg** | ^b^**NTP** | ^c^**NFN** | ^d^**NFP** | ^e^**NTN** | **precision** | **recall** | **specificity** | ^f^**ROC_AUC** | ^g^**PR_AUC** | ^h^**MCC** | ^i^**F2** |
| --- | --- | --- | --- | --- | --- | --- | --- | --- | --- | --- | --- |
| **^j^GB** |  |  |  |  |  |  |  |  |  |  |  |
|  | 24 | 4 | 247 | 725 | 0.090 | 0.874 | 0.746 | 0.859 | 0.110 | 0.230 | 0.319 |
| **^k^RF** |  |  |  |  |  |  |  |  |  |  |  |
|  | 24 | 4 | 238 | 734 | 0.092 | 0.865 | 0.755 | 0.857 | 0.106 | 0.232 | 0.323 |
| **^l^Emb** |  |  |  |  |  |  |  |  |  |  |  |
|  | 23 | 5 | 232 | 741 | 0.994 | 0.762 | 0.762 | 0.848 | 0.104 | 0.224 | 0.316 |
| **^m^MLP** |  |  |  |  |  |  |  |  |  |  |  |
|  | 22 | 5 | 216 | 757 | 0.094 | 0.805 | 0.778 | 0.852 | 0.109 | 0.226 | 0.321 |
| **^n^TFIM** |  |  |  |  |  |  |  |  |  |  |  |
|  | 22 | 6 | 226 | 746 | 0.089 | 0.795 | 0.767 | 0.842 | 0.107 | 0.214 | 0.308 |
| **^o^SVC** |  |  |  |  |  |  |  |  |  |  |  |
|  | 22 | 5 | 252 | 720 | 0.082 | 0.803 | 0.741 | 0.824 | 0.094 | 0.201 | 0.290 |
| **^p^LR** |  |  |  |  |  |  |  |  |  |  |  |
|  | 22 | 6 | 246 | 727 | 0.084 | 0.802 | 0.747 | 0.828 | 0.096 | 0.204 | 0.295 |
